# Proteotyping as alternate typing method to differentiate *Campylobacter coli* clades

**DOI:** 10.1038/s41598-019-40842-w

**Published:** 2019-03-12

**Authors:** Matthias Frederik Emele, Sonja Smole Možina, Raimond Lugert, Wolfgang Bohne, Wycliffe Omurwa Masanta, Thomas Riedel, Uwe Groß, Oliver Bader, Andreas Erich Zautner

**Affiliations:** 1 Institut für Medizinische Mikrobiologie, Universitätsmedizin Göttingen, Kreuzbergring 57, 37075 Göttingen, Germany; 2 Department of Food Science and Technology, Biotechnical Faculty, University of Ljubljana, Jamnikarjeva 101, 1000 Ljubljana, Slovenia; 3 Present Address: Department of Medical Microbiology, Maseno University Medical School, Private Bag, Maseno, Kenya; 4 Leibniz-Institut DSMZ-Deutsche Sammlung von Mikroorganismen und Zellkulturen, Braunschweig, Germany; 5 Deutsches Zentrum für Infektionsforschung (DZIF), Standort Hannover-Braunschweig, Braunschweig, Germany

## Abstract

Besides *Campylobacter jejuni*, *Campylobacter coli* is the most common bacterial cause of gastroenteritis worldwide. *C*. *coli* is subdivided into three clades, which are associated with sample source. Clade 1 isolates are associated with acute diarrhea in humans whereas clade 2 and 3 isolates are more commonly obtained from environmental waters. The phylogenetic classification of an isolate is commonly done using laborious multilocus sequence typing (MLST). The aim of this study was to establish a proteotyping scheme using MALDI-TOF MS to offer an alternative to sequence-based methods. A total of 97 clade-representative *C*. *coli isolates* were analyzed by MALDI-TOF-based intact cell mass spectrometry (ICMS) and evaluated to establish a *C*. *coli* proteotyping scheme. MLST was used as reference method. Different isoforms of the detectable biomarkers, resulting in biomarker mass shifts, were associated with their amino acid sequences and included into the *C*. *coli* proteotyping scheme. In total, we identified 16 biomarkers to differentiate *C*. *coli* into the three clades and three additional sub-clades of clade 1. In this study, proteotyping has been successfully adapted to *C*. *coli*. The established *C*. *coli* clades and sub-clades can be discriminated using this method. Especially the clinically relevant clade 1 isolates can be differentiated clearly.

## Introduction

Intact cell mass spectrometry (ICMS) emerged as the standard method for the identification of microbial species in clinical microbiological laboratories^1–3^. In this method, species identification is not based on the analysis of individual biomarkers or mass spectrometric fingerprints, but on a comparison of the mass spectrum with a microbial spectra database^4^ or a database of ribosomal protein sequences taking into account *N*-terminal methionine cleavage^5^. Besides species identification, ICMS allows distinction of subspecies by accurate discrimination based on strain specific biomarkers^6^. It has also been demonstrated that MALDI-TOF MS facilitates the classification of unknown bacterial isolates, based on similarities in the mass spectra of these bacterial isolates with protein biomarker databases, also known as phyloproteomics^7^. Mass spectrometry-based typing methods, generally referred to as proteotyping^8^, have been used for about two decades for the characterization of tissues^9^, individual proteins^10^, microbial communities^11^, viruses^12^ and, as already mentioned, bacteria. Among others, mass spectrometry (MS) fingerprinting has already been successfully used for subtyping of methicillin-resistant *Staphylococcus aureus* lineages^13^, *Clostridioides difficile* PCR ribotypes^14^, Shiga-toxigenic *Escherichia coli* strains^15^, *Listeria monocytogenes* lineages^16^, and *Salmonella* serotypes^17^. In previous studies we have, for example, shown that it is possible to discriminate *Salmonella enterica* ssp. *enterica* serovar Typhi from non-typhi serovars which cause less severe gastrointestinal infections^18^. Also we have shown that it is possible to discriminate different sequence types of *Campylobacter jejuni* ssp. *jejuni* by analyzing isoforms of L32-M^19^. These strain-specific characteristics form the basis for the development of a novel microbial typing method that we initially named Mass Spectrometry-based PhyloProteomics (MSPP)^20,21^, which we will, in accordance with the terminology now used in the scientific community^8^, refer to as proteotyping, as our method refers to a limited number of biomarkers and not to all the proteins present in the sample. At the core of the method of proteotyping is an amino acid sequence list of all isoforms that have evolved through non-synonymous mutations in the biomarker genes. These isoforms can be recognized as mass shifts in a superposition of calibrated MALDI-TOF spectra. For each bacterial isolate to be typed, the proteotyping scheme can be used to derive a combination of amino acid sequences from the detected biomarker masses. The functionality of this approach was proven by comparison of proteotyping to the current gold standard multilocus sequence typing (MLST)^22^. The advantage of proteotyping over whole spectrum clustering approaches is that only mass changes associated with a particular set of allelic isoforms of the same protein are considered for phylogeny derivation. Other methods take into account the presence or absence of individual masses as well as peak intensity, what delivers less accurate results^20^. Proteotyping provides further advantages in comparison to common subtyping methods like MLST, ribosomal MLST (rMLST) or whole-genome MLST (wgMLST). MLST has the problem of combining sufficiently variable genes into a typing scheme in order to map phylogenetic relationships^23^. Another disadvantage is that it only considers sufficiently variable core genes, whereas hypervariable, transposable gene sites and the entire genome sequence are not considered^24^. Even well-established whole genome sequencing-based MLST schemes are very expensive and time-consuming^25–27^. Therefore, these methods are not used in everyday clinical routine diagnostics and subtyping of microorganisms is currently restricted to a limited cohort, mostly in epidemiological surveys. In the light of the above, a fast and precise subtyping method like proteotyping enables the conduction of numerous experiments that involve the determination of phylogenetic relatedness.

Besides *C*. *jejuni*, *C*. *coli* is the most common bacterial cause of gastroenteritis worldwide^28,29^. The housekeeping genes of *C*. *jejuni* and *C*. *coli* exhibit 86.5% sequence identity^30^, similar to that observed between the enteric bacteria *E*. *coli* and *S*. *enterica*, which are well studied and thought to have diverged 120 million years ago^31^. *C*. *coli* can be subdivided into three genetic clades, which differ in various ways. Clade 1 isolates of *C*. *coli* are most frequently isolated from farm animals and clinical stool samples of humans suffering from acute diarrhea, whereas clade 2 and clade 3 strains, which are more closely related to each other, are mainly found in environmental waters and samples from waterfowl^32–35^. In a previous study, Sheppard and coworkers showed, that all of the examined cases of human *C*. *coli* infection were caused by lineages belonging to clade 1^33^. Clade 1 is further subdivided into two clonal complexes: ST-828, which makes up 70.5% of the *C*. *coli* isolates, and ST-1150, which makes up 4.5% of *C*. *coli* isolates, whereas clades 2 and 3 do not exhibit a clonal complex substructure^33^. An examination of the divergence in *C*. *jejuni* estimated the speciation of *C*. *jejuni* and *C*. *coli* to have occurred 6580 years ago and clonal complex sub-structuring even more recently^36^. For the maintenance of the three *C*. *coli* clades, gene pools of these clades have to be kept separate. A simple explanation for how these gene pools are kept separate would be through a general reduction in the overall level of recombination by recombinational barriers, but as previously mentioned, there is frequent recombination within each clade^33^. In principle, three kinds of recombinational barriers can be described. The first kind of recombinational barrier that enables the maintenance of the *C*. *coli* clade system are mechanistic barriers, which are imposed by the homology dependence of recombination^37^ or other factors, like modification and restriction systems^38^. The second kind of recombinational barriers are ecological barriers, meaning a physical separation of bacterial populations in distinct niches. The third are adaptive barriers, describing a selection against hybrid genotypes^39^. Subtypes belonging to *C*. *coli* clade 1 numerically dominate in clinical samples. It is possible that there are genomic differences affecting the pathogenicity of *C*. *coli* clade 1 isolates but these differences are not required to explain the overrepresentation of this clade in human samples as isolates of this clade plainly dominate in disease reservoirs and food chain sources^33^. Comparative analysis of *C*. *coli* clades suggests that potential virulence factors and resistance mechanisms are not restricted to a single clade. Genes encoding proteins involved in chemotaxis and capsule formation were observed in different clades of *C*. *coli*^40^. The clustered regularly interspaced short palindromic repeat (CRISPR) locus, which is considered to serve as prokaryotic immune system and protection against invasion of alien genetic elements is also present in all *C*. *coli* clades, although its genomic location differs^41,42^. Also, the cytolethal distending toxin (*cdt)* genes are reported to be ubiquitous in all *C*. *coli* strains^43–46^. The *cdt* genes are well conserved in *C*. *coli*, although size and sequences of the respective genes do vary between strains^47^.

In this study, we have established a proteotyping scheme for subtyping of *C*. *coli* isolates. *C*. *coli* isolates from different sources were MLST-typed and therewith it was shown that our test cohort included isolates of all three established clades and subclades. These isolates were typed by ICMS/proteotyping and their phyloproteomic relatedness was deduced. Comparison of the obtained phyloproteomic proteotyping-based unweighted pair group method with arithmetic mean (UPGMA) tree with the corresponding MLST-based UPGMA dendrogram demonstrated that proteotyping is able to differentiate the clinically relevant clade 1 isolates from clade 2 and 3 isolates.

## Results and Discussion

**Figure 1:**
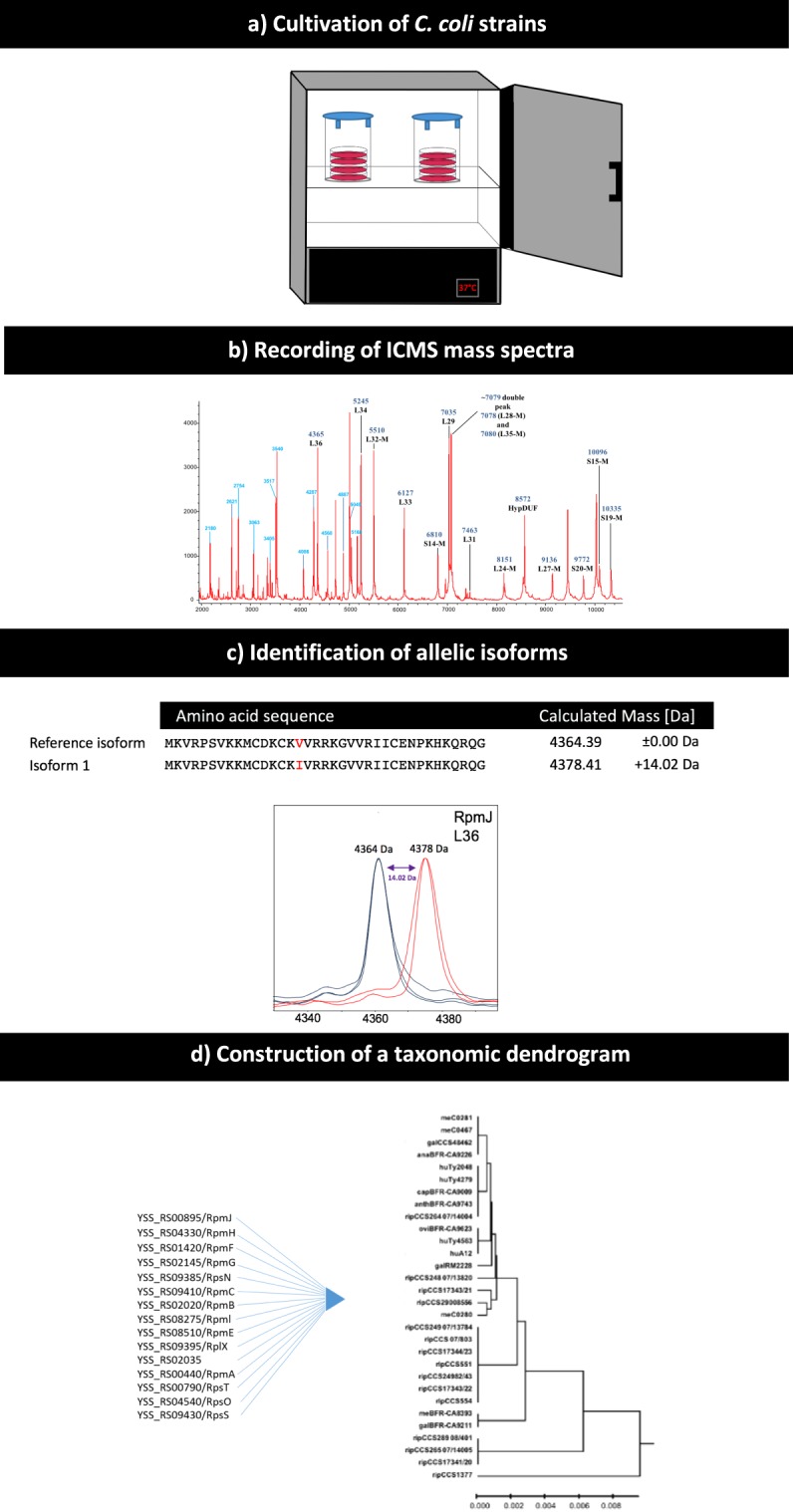
Proteotyping workflow (**a**) Culturing *C*. *coli* strains under microaerophilic conditions. (**b**) Recording of MALDI-TOF mass spectra. (**c**) Designation of allelic isoforms by comparison of mass spectra of all measured *C*. *coli* strains with the allelic isoform list established on the basis of sequence data available in the wgMLST and rMLST databases. (**d**) Concatenation of the amino acid sequences of the identified isoforms into a single continuous sequence and calculation of a taxonomic dendrogram (UPGMA).

Previously, we have established a standard workflow for setting up a new proteotyping (MSPP) scheme and a proteotyping procedure^20^. Following this workflow for *C*. *coli*, (i) we recorded a mass spectrum of the genome sequenced *C*. *coli* reference strain RM2228 (ATCC BAA-1061) and assigned ICMS spectrum masses to open reading frames; (ii) we have compiled a collection of allelic isoforms of the assignable spectrum masses by analyzing the total 1,565 *C*. *coli* sequence datasets deposited in the wgMLST and rMLST databases. Accordingly, we were able to calculate a frequency distribution of the individual allelic isoforms based on these 1,565 *C*. *coli* genomes (Supplementary Table 2). According to the proteotyping scheme (Fig. 1), the spectra of the 97 cultured *C*. *coli* isolates were recorded, following pre-processing and calibration. Mass shifts in comparison to the *C*. *coli* reference strain RM2228 were estimated and the allelic isoforms were assigned by matching of the measured biomarker mass with the calculated masses from the isoform database set. A phyloproteomic proteotyping-based UPGMA-tree was calculated after fusing the amino acid sequences of all biomarker ions included in the *C*. *coli* proteotyping scheme for each tested isolate.

### Identification of biomarker ions

**Figure 2:**
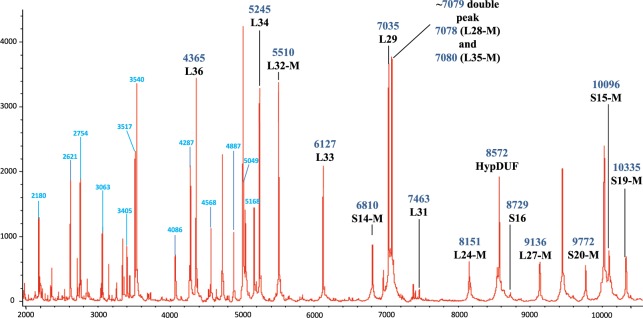
Mass spectrum of the genome sequenced *C*. *coli* reference strain RM2228. Singularly charged biomarker ions identified by comparison of measured molecular masses with calculated masses based on the reference genome are marked in black, doubly/multiply charged ions are labeled in blue, and two so far not identified biomarker ions are labeled with a question mark “?”. The peak at *m/z* ≈ 7,079 corresponds to a fused double peak of biomarkers L28-M (*m/z* = 7,078) and L35-M (*m/z* = 7,080). In *C*. *coli* isolates of the MLST-Clade 3, there is an allelic isoform for L28-M, which has a molecular weight 16 Da lower than the mass of L35-M and therefore two single peaks for L28-M and L35-M can be registered instead of the double peak (see Fig. 3).

**Figure 3:**
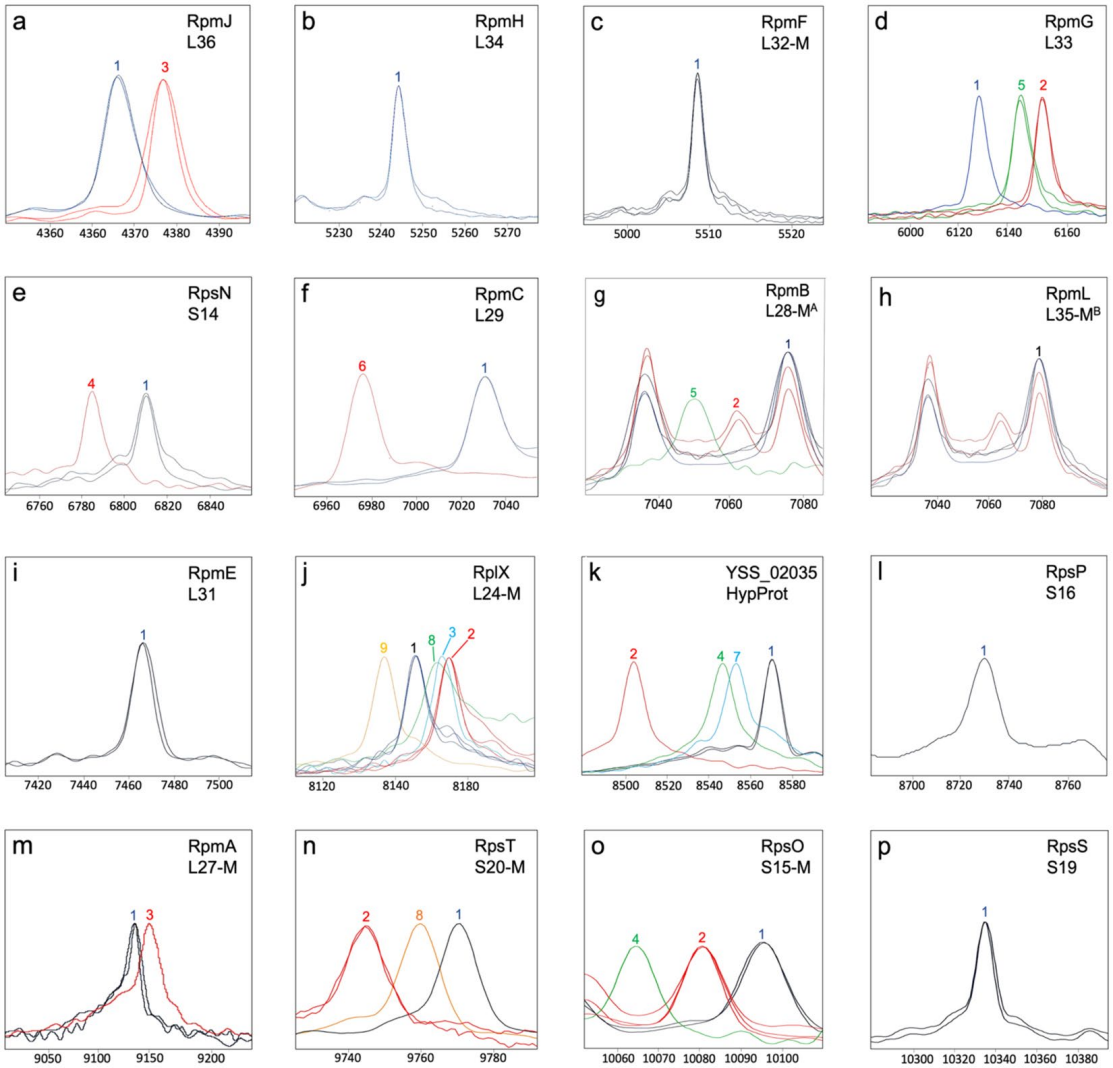
*C*. *coli*-specific proteotyping biomarkers (**a**–**o**). Spectra of representative *C*. *coli* strains were superimposed to illustrate the mass differences between allelic isoforms detected in our *C*. *coli* collection. X-Axis: mass [Da] charge-1 ratio, scale 200 Da. Y-Axis: intensity [10x arbitrary units], spectra were individually adjusted to similar noise level for better visualization of low-intensity peaks. Color codes: the isoform of *C*. *coli* reference strain RM4661 is illustrated in blue; red, light green, dark green, purple and orange are further isoforms. Isoforms lacking *N*-terminal methionine are appended with “-M”. ^A^(**g**) The peak at *m/z* ≈7,079 is a superposition of the biomarker ion masses L28-M (*m/z* = 7,078) and L35-M (*m/z* = 7,080). In contrast, the allelic isoforms 2 and 3 (−14 Da and −28 Da, respectively) are mere L28-M peaks. ^B^(**h**) For the biomarker L35-M we could only detect one allelic isoform in our test cohort, which is superimposed by the biomarker mass L28-M in the spectrum of *C*. *coli* RM2228. In order to show the not superimposed L35-M peak in h an additional spectrum of a clade 3 *C*. *coli* isolate was added, in which the L28-M peak is shifted by −14 Da and therefore L35-M is not superimposed.

With reference to the genome sequence of the *C*. *coli* strain RM2228, 16 single charged biomarker masses, in the range of 4,000 and 10,500 *m/z*, were associated to a specific gene (Figs 2 and 3). The standard deviation for a measurement representing a sum of 6 recordings was less than 0.8 Da and the difference between measured mass and calculated average mass was at maximum 1.35 Da (Supplementary Table 3). The identified biomarkers were RpmJ (L36; 4365 Da), RpmH (L34; 5245 Da), RpmF (L32-M; 5510 Da), RpmG (L33; 6127 Da), RpsN (S14-M; 6810 Da), RpmC (L29; 7035 Da), RpmB (L28-M; 7078 Da), RpmI (L35-M; 7080 Da), RpmE (L31; 7463 Da), RplX (L24-M; 8151 Da), hypothetical protein DUF465 (Cj0449c homologue; 8572 Da), RpsP (S16; 8729 Da), RpmA (L27-M; 9136 Da), RpsT (S20-M; 9743), RpsO (S15-M; 10096 Da), and RpsS (S19-M; 10335 Da). The genes of the 16 biomarker proteins included in the *C*. *coli* proteotyping scheme are distributed across the entire genome of strain RM2228, similar to the seven established MLST markers, and are therefore suitable for the derivation of phylogeny.

These 16 biomarkers are generally identical to those in the proteotyping scheme of *C*. *jejuni* ssp. *jejuni* and *C*. *jejuni* ssp. *doylei*^20,21^. Differences were that in case of RpsU (S21; 9140.9 Da), RpsQ (S17; 9591.5 Da), and RplW (L23; 10554.3 Da), as well as in case of their de-methioninated isoforms, no visible peak could be detected in any of the examined *C*. *coli* strains. Therefore, these three biomarkers were not included in the current *C*. *coli* proteo-typing scheme.

In contrast to *C*. *jejuni* ssp. *doylei*, the biomarker L22-M could be detected in the *C*. *coli* mass spectrum and therefore included in the scheme. L22-M was de-methioninated as in the mass spectrum of *C*. *jejuni* ssp. *jejuni*.

The *N*-terminal methionines of the biomarkers S14-M, S20-M, L24-M, and L32-M were cleaved off in *C*. *coli* as well as in *C*. *jejuni* ssp. *jejuni* and *C*. *jejuni* ssp. *doylei*.

However, five differences were found with respect to the posttranslational modification of the biomarkers by proteolytic removal of the *N*-terminal methionine: In comparison to *C*. *jejuni* ssp. *jejuni*, the *N*-terminal methionine of the biomarker ions S15, S19, L28, and L35 is removed in *C*. *coli*, which is also the case with *C*. *jejuni* ssp. *doylei*^21^.

As with *C*. *jejuni* ssp. *jejuni*, but in contrast to *C*. *jejuni* ssp. *doylei*, the *N*-terminal methionine of L27 remains attached in *C*. *coli*.

Since all five differences were observed in each case for all isolates of the different *Campylobacter* species or sub-species, this confirms the findings of Fagerquist and coworkers that the post-translational modifications are species- and sub-species-specific but not isolate-specific^48^. Accordingly, one can distinguish the three *Campylobacter* species or sub-species solely on the basis of the presence or absence of the *N*-terminal methionine of L27 and S15, S19, L28, or L35.

### Establishment of an allelic isoform list

In the next step, we compiled a collection of allelic isoforms of each of the 16 biomarkers of the *C*. *coli* proteotyping scheme. For this purpose, we used the 1,565 *C*. *coli* genome sequences available in the wgMLST and rMLST databases.

The gene sequence deposited for each biomarker isoform was translated into an amino acid sequence and aligned. Subsequently, the molecular mass for each individual isoform was calculated. Between 3 and 9 isoforms for each biomarker ion could be identified within the data received from the rMLST and wgMLST databases. The frequency of occurrence of isoforms varied from >99% to a single occurrence of the isoform, where in cases of single occurrences in the rMLST and wgMLST databases, a sequencing error must also be considered. For each of the 16 biomarkers, at least two isoforms with a relative increased frequency were found in the database, which means that these masses can serve as phylogenetic discriminators (Supplementary Table 2).

### MLST Typing of a microbial isolate collection

To validate the *C*. *coli* proteotyping scheme a cohort of 101 isolates (*C*. *coli* reference strain RM2228, 96 *C*. *coli* Isolates, and 4 *C*. *jejuni* isolates) was typed by both MLST and proteotyping. The isolates were chosen in such a way that all clades and sub-clades were represented. According to the MLST results, 83 isolates belonged to clade 1. Out of these clade 1 isolates, six belonged to the sub-clade 1B and two further belong to sub-clade 1 C, while the remaining 75 isolates formed sub-clade 1A (ST828). These clade 1 isolates were mainly isolated from human faeces (19), and food-associated samples like chicken meat (21), waterfowl (7), turkey meat (6), swine meat (6), and cattle (5). But only four isolates originated from environmental water. Seven isolates, originating from environmental water, belonged to clade 2, and three isolates also originating from environmental water belonged to clade 3. Additionally, we included four isolates outside the defined MLST clades, but also identified as *C*. *coli* by conventional MALDI-TOF MS. MLST results of three of these four isolates meC0280 (ST6994), mecC0281 (ST6992), and meC0467 (ST6993) originating from turkey cloacal swabs suggested a closer relationship to *C*. *jejuni* and the fourth isolate CCS1377 (ST7908), an environmental water isolate, formed a separate clade in between clade 2 and clade 3 (Supplementary Fig. 1).

### Identification of allelic isoforms

Measurements of the isolates of the study cohort were performed in the same way as for the reference strain *C*. *coli* RM2228. Allelic isoforms were identified by comparison of the masses of candidate allelic isoforms to the reference spectrum of *C*. *coli* RM2228 and by matching the mass differences with the isoform list. For isoforms with the same mass difference to the reference in RM2228, or more precisely, with the same amino acid substitutions, but at different positions in the amino acid sequence, additional DNA sequencing was done using the primers listed in Table 1.

**Table 1:** Oligonucleotide primers used for sequencing of the *C*. *coli* biomarker genes included in the proteotyping scheme.

ORF No. (RM4661)	Gene product	Forward primer (5′ → 3′)	Reverse primer (5′ → 3′)	Amplicon length [bp]
YSS_RS00895	RpmJ/L36	AGCTGCTGCTTCATCTTCACT	AGCCTTGATAAAGGGCGTATC	490
YSS_RS04330	RpmH/L34	AAATGCTCGGGCAAATTGATTA	GCCATCGCAATACCACTTTT	512
YSS_RS01420	RpmF/L32	TGCACCACTATGTCCTGCTG	TGCCACAATGCAAGGTTTTGT	728
YSS_RS02145	RpmG/L33	AGCTGATGGCGTTGAAATGG	ACCCCCAACCATCGGATTTG	430
YSS_RS09385	RpsN/S14	ACACGACGACCTGGTTTAGA	TCGGTCTTGATGAGCAGTTGA	611
YSS_RS09410	RpmC/L29	GGTCTGCATTCAACCGCTAC	GCCAAATTGAAGCAGCTCGT	668
YSS_RS02020	RpmB/L28	CGTCAAGTTCATTATGGCGCT	TGGAACAAAATGCCCGTCCA	742
YSS_RS08275	RpmI/L35	GCAAGCAGCATTGATACGCA	GCTTGGCTATTTTGCAAAGGATT	715
YSS_RS08510	RpmE/L31	GCAAGGTTTTTCCTGATGCTGT	TGGCATACCCGCATCACTC	756
YSS_RS09395	RplX/L24	TCGGAACTCGTATCTTTGGGC	CAGGAAAACCTTCACGCACT	578
YSS_RS02035	DUF465	GCTGCTGGGTAAGATTTTGGT	TCGTGTAACCCTAGAAGATGGC	584
YSS_RS00440	RpmA/L27	AGTTAGCGTTGGCGATGAGTT	AACGAAGATGATATCCCCGCC	783
YSS_RS00790	RpsT/S20	GCTCTTCTTCGAGTTTGGGTT	GGTGGATTGGGTGTTATGCT	765
YSS_RS04540	RpsO/S15	ATATCGGATACAACCGCGCA	GCATACTCGCTAGCTTTGGT	636
YSS_RS09430	RpsS/S19	AGCACCAGCATCTACACGAC	ATGGCAAGTATCGGCGAAGT	782

Within this study population, we detected five isoforms for RplX (L24-M) and four isoforms for protein DUF465. Three isoforms each were detected for RpmG (L33), RpmB (L28-M), RpsT (S20-M), RpsO (S15-M) and two isoforms each for RpmJ (L36), RpsN (S14-M), RpmC (L29) and RpmA (L27-M). For RpmH (L34), RpmF (L32-M), RpmI (L35-M), RpsP (S16) and RpsS (S19-M) only one isoform was detected (Fig. 3, Supplementary Table 2).

### Computing of a phyloproteomic UPGMA-dendrogram

The amino acid sequences of the 16 identified biomarker isoforms were concatenated to one continuous sequence for each isolate, which was in turn used to compute a phyloproteomic tree by conventional clustering algorithms (UPGMA).

**Figure 4:**
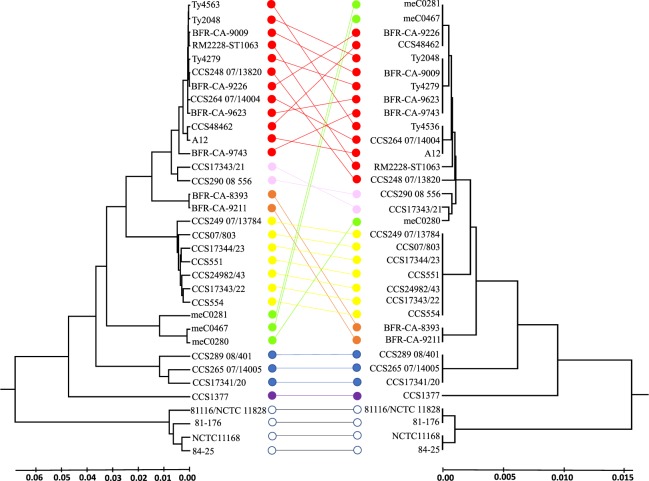
Comparison of MLST-based and proteotyping-based UPGMA dendrograms. The MLST-based phylogenetic tree (left) as well as the proteotyping-based dendrogram (right) were constructed by UPGMA. The MLST dendrogram resulted from 7 loci, the proteotyping-based dendrogram from the amino acid sequences of 16 identified biomarker ions. The different *C*. *coli* clades and sub-clades are represented by different colors. In addition, four *C*. *jejuni* isolates have been included in the illustration, which form their own *C*. *jejuni* clade. Color codes: clade 1A – red, clade 1B – pink, clade 1C – orange, clade 2 – yellow, clade 3 – blue, isolate CCS1377 – purple, isolates meC0280, mecC0281, and meC0467 – green, *C*. *jejuni* isolates – white. Lines connect the corresponding isolates in the different trees. As it can be seen, there are only crossings of connecting lines within one clade (corresponding to one color), whereas different colors (clades) do not cross each other. This demonstrates that proteotyping can be used to distinguish the clades clearly from each other. The only exceptions are the three isolates meC0280, mecC0281, and meC0467 labeled in green. These form their own clade in the MLST-based tree (Supplementary Fig. 1), but in the core genome alignment (Supplementary Fig. 2) they cluster with *C*. *coli* clade 1. This means that for isolates of this group the proteotyping-based tree is similar to a core genome alignment, while MLST is less suitable.

Within our test cohort, the combined amino acid sequences in our collection yielded 12 (plus two for *C*. *jejuni*) different proteotyping-based sequence types. For an evaluation of the constructed proteotyping-based UPGMA-tree, an MLST-based UPGMA-tree was computed for comparison. This was done with 30 *C*. *coli* isolates and 4 *C*. *jejuni* isolates representative of all MLST clades and sub-clades as well as all 12 proteotyping-derived types. For clarity, the complete test cohort was reduced from 101 isolates to 34 representative isolates. The UPGMA-tree deduced from the concatenated biomarker protein sequences was generally concordant with MLST results (Fig. 4).

The *C*. *coli* proteotyping scheme was clearly able to distinguish *C*. *jejuni* and *C*. *coli* isolates. Since the three biomarkers RpsU/S21, RpsQ/S17, and RplW/L23 were not detectable in the *C*. *coli* mass spectrum, the *C*. *coli* proteotyping scheme had to be reduced by these three biomarkers, which nevertheless still allows sufficient differentiation between the two microbial biospecies. As already stated above, it is feasible to distinguish both microbial species solely on the basis of the presence or absence of the *N*-terminal methionine of the biomarkers of L27 and S15, S19, L28, or L35. In addition, there are allelic isoforms of the biomarkers, which are characteristic for each of the biospecies e.g.: L32-M − T48N; L31 − T23V + A29S + N38S; and S20-M − N41K + G42N (using *C*. *jejuni* NCTC 11168 as reference strain).

Furthermore, the *C*. *coli-*specific proteotyping scheme precisely discriminated isolates belonging to different clades, illustrated by the absence of crossing connection lines of different colors in Fig. 4. All isolates of sub-clade 1A, and of the sub-clades 1B and 1C as well as of clade 2 and 3 form individual clusters. However, only the sub-clades 1A and 1B form neighboring clusters, while the isolates of sub-clade 1C are to be found between the clades 2 and 3.

Besides the isolates representing the well-established clades and sub-clades of *C*. *coli*, four isolates not belonging to either of these clades were included in our study: CCS1377, meC0280, mecC0281, and meC0467.

Isolate CCS1377 is, in both the MLST-based and the proteotyping-based dendrograms, a single isolate placed outside the *C*. *coli* clades, which is evolutionarily more closely related to *C*. *jejuni*.

In contrast, the three isolates meC0280, mecC0281, and meC0467, which form a separate clade in the MLST-based neighbor-joining tree branching off at the basis of the *C*. *jejuni* branch (Supplementary Table 1), did not form a common cluster in the proteotyping-based tree. The isolates mecC0281 and meC0467 clustered together with the clade 1 A isolates, in contrast meC0280 clustered together with the isolates of sub-clade 1B. Using a whole genome neighbor-joining parsnp algorithm as reference we could demonstrate that the isolates meC0280, mecC0281, and meC0467 integrate into the cluster of clade 1 *C*. *coli* isolates (Supplementary Fig. 2). Therefore, the clustering in the proteotyping-based UPGMA-tree corresponds more closely to the clustering of the whole genome neighbor-joining parsnp-tree. Here proteotyping proves to be a sufficient differentiation tool that seems superior to 7-gene MLST-based phylogeny.

In summary, our proteotyping scheme clearly differentiates the clinically relevant clade 1 isolates from the other clades. If this scheme would be integrated into a subtyping module of the mass spectrometry evaluation software, we would be able to determine the clade and the clinical relevance of an isolate as early as in the mass spectrometric species determination phase.

## Materials and Methods

### *Campylobacter coli* and *Campylobacter jejuni* isolates

A total of 101 *Campylobacter* isolates were included in the presented study. Of these were 97 *C*. *coli* Isolates including 21 isolates from chicken, 19 from human feces (clinical isolates of patients with campylobacteriosis), 15 from environmental water, 9 from turkey, 7 from water fowl, 6 from swine, 5 from cattle, 3 from wild bird, 3 from sheep, 2 from goat feces, 2 from ape feces, 2 from wild boar, and one from deer, bivalves and Eurasian otter. Twenty four of these isolates (including all 15 riparian and 9 chicken isolates) were provided by the Department of Food Science and Technology, at University of Ljubljana, Slovenia; 54 isolates (animal isolates) were provided by the German *Campylobacter* Reference Center of the Bundesinstitut für Risikobewertung (Federal Institute for Risk Assessment) in Berlin, Germany; 19 isolates (human isolates) originated from stool samples of suspected campylobacteriosis patients treated at the University Medical Center Göttingen, Germany. The genome-sequenced *C*. *coli* reference strain, RM2228, as well as the four *C*. *jejuni* reference strains NCTC 11168, 81–176, 84–25, and 81116/NCTC 11828 were obtained from the National Collection of Type Cultures (NCTC), Salisbury, UK, Manassas, Virginia, USA. The isolates, especially the subset for Fig. 4, were picked so that the test collection represented a high genetic diversity. Initial species identification was performed using the MALDI Biotyper system (Bruker Daltonics, Bremen, Germany). Results with MALDI Biotyper identification score values ≥ 2.000 were assessed as correct. Additionally, the well-established multiplex polymerase chain reaction of Vandamme and coworkers was used to distinguish between *C*. *jejuni* and *C*. *coli*^49^.

### Bacterial culture

*C*. *coli* and *C*. *jejuni* strains were stored for long-term storage in Cryobank tubes at −80 °C (Mast Diagnostica, Reinfeld, Germany). For the experiments, they were incubated as one batch overnight under microaerophilic conditions (5% O_2_, 10% CO_2_, 85% N_2_) on Columbia agar base (Merck, Darmstadt, Germany) supplemented with 5% sheep blood (Oxoid Deutschland GmbH, Wesel, Germany). Experiments were carried out under biosafety level 2 conditions.

### The preparation of a matrix solution containing human insulin

To prepare the matrix solution, the matrix substance, purified with α-cyano-4-hydroxy-cinnamic acid (HCCA; Bruker Daltonics, Bremen, Germany), was dissolved in the standard solvent consisting of acetonitrile 50%, water 47.5% and trifluoroacetic acid 2.5%. The resulting concentration was 10 mg HCCA/mL. Recombinant human insulin (Sigma-Aldrich, Taufkirchen, Germany) in HCCA solution was added to serve as an internal calibrant for spectrum evaluation. The final concentration of human insulin in 50% aqueous acetonitrile was 10 pg/μL. The exact determination of the insulin peak mass was carried out experimentally by mixing with the Bruker Test Standard and consecutive recording of mass spectra. The insulin peak was detected at an *m/z* = 5,806.1. The insulin peak functioned as an internal calibrant for all *C*. *coli* mass spectra. Insulin proved to be particularly suited, because its mass did not coincide with other recorded biomarker masses. The use of an internal calibrant significantly increases precision in the determination of biomarker mass changes. With this approach, we were able to detect mass differences with a standard deviation of less than 1 Da.

### Recording MALDI-TOF mass spectra

The preparation of the samples used in MALDI-TOF MS was carried out in two variants: by smear preparation and extraction. Five colonies of an overnight agar plate culture were harvested for the preparation of the extract samples and then given into 300 μL double distilled water. The colonies were suspended by rigorous mixing. Subsequently, 900 μL absolute ethanol was added and the suspension was thoroughly mixed by repeated up-and-down pipetting. After complete suspension of the bacterial cells, the suspensions were centrifuged for 1 minute at 13,000 × g. Subsequently, the supernatant was discarded and the pellets dried at room temperature for 10 minutes. By vortexing during the drying process, the pellet was thoroughly resuspended in 50 μL of 70% formic acid. In the next step, 50 μL acetonitrile was added to each tube and mixed with the pipette, followed by centrifugation of the mixture at 13,000 × g for 2 min. After centrifugation, 1 μL of the supernatant was pipetted onto a sample position on a polished steel MALDI target plate, and was left to dry for about 5 minutes at room temperature. Subsequently, each sample position was coated with 1 μL of the HCCA matrix containing the internal calibrant, human insulin. Again, the matrix-coated target was left to dry at room temperature. Once the matrix had dried, the samples were ready for mass spectrometric measurement^50^. Recording of the mass spectra was performed according to the standard recommendations for the MALDI Biotyper System (Bruker Daltonics, Bremen, Germany). Six hundred spectra in a mass range of 2–20 kDa were recorded in 100-shot steps on an Autoflex III system and summed up. Only if the MALDI Biotyper identification score values were ≥2,000 they were judged to be valid.

### Assignment of specific allelic isoforms to biomarker ions in mass spectra

Analysis of mass spectra was performed using FlexAnalysis and the algorithms implemented therein (Bruker Daltonics, Bremen, Germany). First, the spectra were calibrated internally to the set insulin peak (*m/z* = 5,806.1), followed by subsequent pre-processing, baseline subtraction and smoothing. The theoretical average molecular weight of the proteins that correspond to any open reading frame (see Supplementary Table 3) was derived from the amino acid sequence using the molecular weight calculator in the ExPASy Bioinformatics Resource Portal (http://web.expasy.org/compute_pi/). It is important to note that posttranslational modifications occasionally occur in ribosomal proteins of *Enterobacteriaceae*. For this reason, further optional molecular weights had to be considered for each open reading frame^51^. Plausible post-translational modifications are proteolytic removal of *N*-terminal initiator methionine (iMet) which was considered to result in a mass difference of −131 Da, *N*-terminal acetylation^52,53^ and the presence or absence of disulfide bonds for example in the calibrant human insulin.

The unambiguous naming of biomarker masses, i.e. the assignment of a biomarker peak to a specific allelic isoform, was done by comparing the measured masses with the calculated masses from the reference *C*. *coli* RM2228 genome. If there was no clear correspondence between a biomarker mass in the recorded mass spectrum of a specific isolate in the test cohort to the mass calculated from the *C*. *coli* RM2228 reference genome, the biomarker mass identification was done by matching the measured biomarker mass to calculated masses in entries of the ribosomal MLST (rMLST) database or the whole genome MLST (wgMLST) database, respectively. If still no unambiguous matching was found for the biomarker mass, the mass spectrum was examined for peaks with biomarker masses that correspond to possible mass shifts due to mutations in the original biomarker resulting in amino acid exchanges (Supplementary Table 2).

Every recorded allelic isoform in the test cohort was reconfirmed by amplification via PCR and consecutive Sanger sequencing of the obtained amplicons (Seqlab, Göttingen, Germany). All primers used in the experiment are listed in Table 1. The parameters of the PCR reactions were set as follows: pre-denaturation at 94 °C for 300 sec; denaturation at 94 °C for 30 sec; annealing at 55 °C for 30 sec; elongation at 72 °C for 30 sec; repetition for 30 amplification cycles; post-elongation at 72 °C for 600 sec. In each of the isolates studied, the predicted mutations were found in the genes encoding the corresponding biomarker protein, which proved the identities of the peaks. Both, nucleotide and amino acid sequences of the allelic isoforms of biomarkers newly described during the study, have been deposited at the Genbank. The accession numbers of all biomarkers (nucleotide and amino acid sequences) are listed in Supplementary Table 4. MLST sequence types of all isolates analyzed in the study have been deposited at the *Campylobacter* MLST database (https://pubmlst.org/campylobacter/).

### Calculation of phylogenetic and phyloproteomic dendrograms

The Molecular Biology and NGS Analysis Tool Geneious V11.1.2 (http://www.geneious.com) was used to translate and align the protein sequences taken from the rMLST and wgMLST databases. Additionally, Geneious was used to trim and align sequences from confirmatory sanger sequencing^54^.

Calculation of the MLST- and proteotyping-based UPGMA-dendrogram was done with the help of the MEGA7 software^55^. For the assignment of MLST sequence types and clonal complexes, the *C*. *coli*/*C*. *jejuni* MLST website (https://pubmlst.org/campylobacter/) was consulted^56^. The evolutionary history was inferred using the Neighbor-joining method^57^. The evolutionary distances were computed using the Maximum Composite Likelihood method^58^. Core-genome alignments were computed using Parsnp and FastTree2^59^ was used to calculate the maximum-likelihood (ML) phylogenetic tree. Parsnp and FastTree2 are both implemented in the Harvest package^60^.

### Ethical Approval

Ethical approval for the study was obtained from Ethics Commission of the University Medical Center Göttingen, Germany. No humans, animals, or personalized data were used for this study.

## Supplementary information

Supplementary information to: Proteotyping as alternate typing method to differentiate Campylobacter coli clades
