# Supplementary material for: Proteotyping as alternate typing method to differentiate Campylobacter coli clades
Source: Sci Rep. 2019 Mar 12;9:4244. doi: 10.1038/s41598-019-40842-w (PMC6414644; doi:10.1038/s41598-019-40842-w)
Supplement: Supplementary file 1 — Supplementary information to: Proteotyping as alternate typing method to differentiate Campylobacter coli clades [file 41598_2019_40842_MOESM1_ESM.pdf]

Supplementary information to:

Proteotyping as alternate typing method to differentiate *Campylobacter coli* clades

Matthias Frederik Emele[1], Sonja Smole Možina[2], Raimond Lugert[1], Wolfgang Bohne[1], Wycliffe Omurwa Masanta[1,3], Thomas Riedel[4,5], Uwe Groß[1], Oliver Bader[1], Andreas Erich Zautner[1§]

[1]Institut für Medizinische Mikrobiologie, Universitätsmedizin Göttingen, Kreuzbergring 57, 37075 Göttingen, Germany

[2]Department of Food Science and Technology, Biotechnical Faculty, University of Ljubljana, Jamnikarjeva 101, 1000 Ljubljana, Slovenia

[3]Current address: Department of Medical Microbiology, Maseno University Medical School, Private Bag, Maseno, Kenya.

[4]Leibniz-Institut DSMZ-Deutsche Sammlung von Mikroorganismen und Zellkulturen, Braunschweig, Germany

[5]Deutsches Zentrum für Infektionsforschung (DZIF), Standort Hannover-Braunschweig, Braunschweig, Germany

[§] Corresponding author: Andreas E. Zautner

Universitätsmedizin Göttingen

Institut für Medizinische Mikrobiologie

Kreuzbergring 57

D-37075 Göttingen, Germany

Phone: +49-551-398549

FAX: +49-551-395861

E-mail: azautne@gwdg.de

Supplementary Tables

Supplementary Table 1. List of all *C. coli* and *C. jejuni* isolates included in the study

No.	Isolate	Year of isolation	Origin	MLST-ST	asp	gln	glt	gly	pgm	tkt	unc	Clade
1	BFR-CA-6144	2007	Wild pheasant, Meat	829	33	39	30	82	113	43	17	1A
2	BFR-CA-7268	2009	Bivalvia	855	33	39	30	79	104	35	17	1A
3	BFR-CA-8266	2010	Boar	1096	33	38	30	82	104	35	17	1A
4	BFR-CA-8393	2010	Turkey, Egg	7018	114	195	103	115	459	164	79	1C
5	BFR-CA-8905	2010	Pig, Liver	854	33	38	30	82	104	43	17	1A
6	BFR-CA-8971	2010	Eurasian otter	829	33	39	30	82	113	43	17	1A
7	BFR-CA-8980	2010	Deer	855	33	39	30	79	104	35	17	1A
8	BFR-CA-9124	2011	Turkey, Appendix	1055	33	39	30	82	104	47	17	1A
9	BFR-CA-9211	2011	Chicken egg, Shell	4148	114	195	103	140	459	164	79	1C
10	BFR-CA-9317	2011	Ape, Faeces	827	33	39	30	82	104	56	17	1A
11	BFR-CA-9614	2011	Milk	827	33	39	30	82	104	56	17	1A
12	BFR-CA-9625	2011	Pig, Kidney	1056	32	39	30	82	104	43	36	1A
13	BFR-CA-9707	2011	Chicken, Heart	6825	33	39	30	116	113	47	17	1A
14	BFR-CA-9965	2011	Chicken, Liver	860	33	39	30	79	113	47	17	1A
15	BFR-CA-10206	2012	Pig, Meat	2869	33	38	44	82	104	44	17	1A
16	BFR-CA-10225	2012	Goose, Meat	1769	33	176	30	115	113	43	17	1A
17	BFR-CA-10258	2012	Duck, Meat	5162	33	66	30	79	113	43	17	1A
18	BFR-CA-10460	2012	Steer	1591	33	39	44	82	118	35	36	1A
19	BFR-CA-10470	2012	Turkey, Liver	860	33	39	30	79	113	47	17	1A
20	BFR-CA-10623	2012	Goat	825	33	39	30	82	113	47	17	1A
21	BFR-CA-10659	2012	Sheep	1750	33	39	30	115	113	43	17	1A
22	BFR-CA-10673	2012	Breeding pigeon	825	33	39	30	82	113	47	17	1A
23	BFR-CA-10733	2012	Turkey, Skin	3990	33	66	30	79	104	43	17	1A
24	BFR-CA-10855	2012	Calf, Faeces	825	33	39	30	82	113	47	17	1A
25	Ty4222	2011	Human faeces	854	33	38	30	82	104	43	17	1A
26	Ty3717	2011	Human faeces	6987	33	38	30	79	104	35	36	1A
27	Ty5430	2011	Human faeces	6988	33	39	30	81	118	44	36	1A
28	Ty3946	2011	Human faeces	825	33	39	30	82	113	47	17	1A
29	Ty5293	2011	Human faeces	854	33	38	30	82	104	43	17	1A
30	Ty4279	2011	Human faeces	901	33	39	30	79	104	43	41	1A
31	Ty2856	2011	Human faeces	6989	53	153	30	78	113	47	17	1A
32	Ty3935	2011	Human faeces	1100	33	39	30	82	104	44	36	1A

33	Ty09380	2011	Human faeces	7017	53	66	30	82	104	47	17	1A
34	Ty4536	2010	Human faeces	1056	33	39	30	82	104	43	36	1A
35	Ty32545	2011	Human faeces	854	33	38	30	82	104	43	17	1A
36	Ty4543	2011	Human faeces	828	33	39	30	82	104	43	17	1A
37	Ty2048	2011	Human faeces	827	33	39	30	82	104	56	17	1A
38	Ty2384	2011	Human faeces	1127	33	153	30	82	104	43	17	1A
39	Ty4191	2011	Human faeces	1172	53	39	44	82	118	35	36	1A
40	Ty5587	2011	Human faeces	854	33	38	30	82	104	43	17	1A
41	Ty5165	2011	Human faeces	1628	33	39	30	79	104	206	17	1A
42	Ty4207	2011	Human faeces	854	33	38	30	82	104	43	17	1A
43	BFR-CA-6266	2007	Quail	829	33	39	30	82	113	43	17	1A
44	BFR-CA-6377	2008	Chicken, Meat	1600	33	39	65	140	104	43	17	1A
45	BFR-CA-6656	2008	Boar, Meat	1680	53	38	30	81	118	225	36	1A
46	BFR-CA-7041	2008	Chicken, Meat	829	33	39	30	82	113	43	17	1A
47	BFR-CA-7417	2009	Swan	855	33	39	30	79	104	35	17	1A
48	BFR-CA-230	2009	Laying hen	3026	33	39	30	82	104	283	41	1A
49	BFR-CA-8320	2009	Piglet, Faeces	854	33	38	30	82	104	43	17	1A
50	BFR-CA-8905	2010	Pig, Liver	854	33	38	30	82	104	43	17	1A
51	BFR-CA-9009	2010	Goat	829	33	39	30	82	113	43	17	1A
52	BFR-CA-9226	2011	Duck, Meat	855	33	39	30	79	104	35	17	1A
53	BFR-CA-9271	2010	Chicken, Liver	1628	33	39	30	79	104	206	17	1A
54	BFR-CA-9310	2008	Goose, Stomach	1628	33	39	30	79	104	206	17	1A
55	BFR-CA-9316	2008	Northern shoveler, Intestine	827	33	39	30	82	104	56	17	1A
56	BFR-CA-9540	2011	Broiler, Skin	828	33	39	30	82	104	43	17	1A
57	BFR-CA-9557	2011	Broiler, Heart	1589	33	39	30	82	189	43	17	1A
58	BFR-CA-9623	2011	Lamb, Meat	6990	32	66	66	82	113	43	17	1(B)
59	BFR-CA-9625	2011	Pig, Kidney	1571	32	39	30	82	104	43	36	1A
60	BFR-CA-9733	2011	Chicken, Meat	832	33	39	30	79	113	43	17	1A
61	BFR-CA-9743	2011	Ape	5150	292	66	30	82	113	206	17	1B
62	BFR-CA-9870	2011	Broiler, Skin	7019	33	39	411	79	113	43	17	1A
63	BFR-CA-10044	2011	Duck, Meat	860	33	39	30	79	113	47	17	1A
64	BFR-CA-10137	2012	Sheep foetus, Stomach	6991	33	38	30	82	104	85	36	1A
65	BFR-CA-10382	2012	Bovine, Meat	827	33	39	30	82	104	56	17	1A
66	BFR-CA-10430	2012	Calf, Faeces	4679	33	38	30	82	113	43	36	1A
67	BFR-CA-10783	2012	Turkey, Appendix	1058	33	39	30	82	104	35	17	1A
68	BFR-CA-10865	2012	Turkey, Skin	1191	33	39	30	82	189	47	17	1A
69	meC0281	2009	Turkey	6992	118	39	30	79	429	3	12	5 (?)
70	meC0467	2009	Turkey	6993	33	39	30	82	113	3	12	5 (?)

71	meC0280	2009	Turkey	6994	33	39	30	79	503	25	294	5 (?)
72	RM2228-ST1063	1998	Chicken	1063	33	39	30	140	113	43	41	1A
73	CCS48462	2008	Chicken, Meat	7852	86	39	30	82	113	332	17	1B
74	CCS48466	2008	Chicken, Meat	7853	33	38	30	82	104	332	36	1A
75	CCS48468	2008	Chicken, Meat	7853	33	38	30	82	104	332	36	1A
76	CCS17341/20	2006	Environmental waters	7907	121	492	451	659	317	208	485	3
77	CCS1377	2008	Environmental waters	7908	334	552	30	241	598	600	471	4(?)
78	CCS248 07/13820	2007	Environmental waters	7855	33	39	30	79	104	35	211	1A
79	CCS52236	2008	Chicken, Meat	7856	33	39	30	82	104	65	17	1A
80	CCS17343/21	2006	Environmental waters	5603	136	188	69	113	270	277	152	1B
81	CCS290 08 556	2008	Environmental waters	7857	135	188	69	113	143	332	152	1B
82	CCS17344/23	2006	Environmental waters	5658	130	240	87	276	335	126	194	2
83	CCS554	2008	Environmental waters	2269	130	86	88	245	288	129	76	2
84	CCS264 07/14004	2007	Environmental waters	7121	32	39	44	82	104	44	17	1A
85	CCS551	2008	Environmental waters	7854	86	528	88	269	755	316	156	2
86	CCS265 07/14005	2007	Environmental waters	7909	121	553	452	602	738	240	183	3
87	CCS29782	2008	Chicken, Meat	7858	33	38	66	115	104	43	17	1A
88	24907/13784	2007	Environmental waters	7859	137	189	174	241	756	126	76	2
89	CCS48467	2008	Chicken, Meat	7853	33	38	30	82	104	332	36	1A
90	CCS52230	2008	Chicken, Meat	3702	33	39	30	82	113	65	17	1A
91	CCS07/803	2007	Environmental waters	7862	398	189	86	269	701	599	153	2
92	CCS33759	2008	Chicken, Meat	3702	33	39	30	82	113	65	17	1A
93	CCS47509	2008	Chicken, Meat	7860	33	38	30	82	118	332	36	1A
94	CCS24982/43	2006	Environmental waters	7863	130	440	370	276	757	129	156	2
95	CCS17343/22	2006	Environmental waters	1981	136	86	88	124	266	238	95	2
96	CCS289 08/401	2008	Environmental waters	7910	121	39	453	660	758	584	470	3
97	A12	2000	Human faeces	7861	130	39	44	82	104	44	68	1B
98	81-176	1989	Human faeces	913	1	2	3	27	5	9	6	*C. jejuni*
99	NCTC11168	1977	Human faeces	267	4	7	40	4	42	51	1	*C. jejuni*
100	84-25	1986	Human, Cerebrospinal fluid	43	2	1	5	3	4	1	5	*C. jejuni*
101	81116/NCTC 11828	1985	Human faeces	185	2	1	1	3	1	1	5	*C. jejuni*

Supplementary Table 2. Overview of all isoforms included in the *C. coli* proteotyping scheme

Locus	Full name / product (ORF Locus tag in RM4661)	calc. average Mass [Da]		Frequency in database
RpmJ/BACT000065; L36; YSS_RS00895				
sequence	MKVRPSVKKMCDKCKVVRRKGVVRIICENPKHKQRQG* (37 aa)			
1*	RM4661 reference isoform	4364.39 Da	±0.00 Da	99.936 % (1564/1565)
2	MKVRPSVKKMCDKCKIVRRKGVVRIICENPKHKQRQG* (V16I)	4378.41 Da	+14.02 Da	0.064 % (1/1565)
3*	MKVRPSIKKMCDKCKVVRRKGVVRIICENPKHKQRQG* (V7I)	4378.41 Da	+14.02 Da	new

RpmH/BACT000063; L34; YSS_RS04330				
sequence	MKRTYQPHGTPRKRTHGFRVRMKTKNGRKVINARRAKGRKRLAV* (44 aa)			
1*	RM4661 reference isoform	5244.28 Da	±0.00 Da	99.550 % (1549/1556)
2	MKRTYQPHGTPRKRTHGFRVRMKTKNGRKMINARRAKGRKRLAV* (V30M)	5276.34 Da	+32.06 Da	0.257 % (4/1556)
3	MKRTYQPHGTPRKRTHGFRVRMKTKNGRKVINARRSKGRKRLAV* (A36S)	5260.28 Da	+16.00 Da	0.193 % (3/1556)

RpmF/BACT000061; L32-M; YSS_RS01420				
sequence	(M)AVPKRRVSKTRAAKRRTHYKVSLPMPIKDKDGSYKMPHRANPNTKEY* (47 aa)			
1*	RM4661 reference isoform	5509.45 Da	±0.00 Da	99.297 % (1554/1565)
2	(M)AVPKRRVSKTRAAKRRTHYKVSLPMPIRDKDGSYKMPHRANPNTKEY* (K29R)	5537.46 Da	+28.01 Da	0.383 % (6/1565)
3	(M)TVPKRRVSKTRAAKRRTHYKVSLPMPIKDKDGSYKMPHRANPNTKEY* (A2T)	5539.47 Da	+30.02 Da	0.128 % (2/1565)
4	(M)VVPKRRVSKTRAAKRRTHYKVSLPMPIKDKDGSYKMPHRANPNTKEY* (A2V)	5537.50 Da	+28.05 Da	0.064 % (1/1565)
5	(M)AVPKRRVSKTRAAKRRTHYEVSLPMPIKDKDGSYKMPHRANPNTKEY* (K21E)	5510.39 Da	+0.94 Da	0.064 % (1/1565)
6	(M)AVPKRRVSKTRAAKRRTHYKVSLPIPIKDKDGSYKMPHRANPNTKEY* (M26I)	5491.41 Da	-18.04 Da	0.064 % (1/1565)

RpmG/BACT000062; L33; YSS_RS02145				
sequence	MRIKVGLKCEECGDINYSTYKNGKNTTEKLELKKYCPRLKKHTLHKEVKLKS* (52 aa)			
1*	RM4661 reference isoform	6126.27 Da	±0.00 Da	90.845 % (1419/1562)
2*	MRIKVGLKCEECGDINYSTYKNSKNTTEKLELKKYCPRLKKHTLHKEVKLKS* (G23S)	6156.29 Da	+30,02 Da	6.017 % (94/1562)
3	MRIKVGLKCEECGDINYSTYKNGKNTIEKLELKKYCPRLKKHTLHKEVKLKS* (T27I)	6138.32 Da	+12,05 Da	1.152 % (18/1562)
4	MRIKVGLKCEECGDINYSTYKNGKNTTEKLELNKYCPRLKKHTLHKEVKLKS* (K33N)	6112.20 Da	-14,07 Da	0.128 % (2/1562)
5*	MRIKVGLKCEECGDINYSTYKNSKNTTDKLELKKYCPRLKKHTLHKEVKLKS* (G23S + E28D)	6142.27 Da	+16.00 Da	1.644 % (26/1562)
6	MRIKVGLKCEECGDINYSTYKNSKNTTEKLELKKYCPRLKKNTLHKEVKLKS* (G23S + H42N)	6133.25 Da	+6.98 Da	0.064 % (1/1562)
7	MRIKVGLKCEECGDINYSTYKNGKNTTEKLELKKYCPKLKKHTLHKEVKLKS* (R38K)	6098.25 Da	-28.02 Da	0.128 % (2/1562)

RpsN/BACT000014; S14-M; YSS_RS09385				
sequence	(M)AKKSMIAKAARKPKFKVRGYTRCQICGRPHSVYRDFGICRVCLRKMGNEGLIPGLKKASW* (60 aa)			
1*	RM4661 reference isoform	6811.24 Da	±0.00 Da	99.361 % (1555/1565)
2	(M)AKKSMIAKAARKPKFKVRAYTRCQICGRPHSVYRDFGICRVCLRKMGNEGLIPGLKKASW* (G20A)	6825.27 Da	+14.03 Da	0.511 % (8/1565)
3	(M)AKKSMITKAARKPKFKVRGYTRCQICGRPHSVYRDFGICRVCLRKMGNEGLIPGLKKASW* (A8T)	6841.27 Da	+30,03 Da	0.128 % (2/1565)
4*	(M)AKKSMIAKAARKPKFSVRGYTRCQICGRPHSVYRDFGICRVCLRKMANEGLIPGLKKASW*(K17S+G48A)	6784.17 Da	-27.07 Da	new

RpmC/BACT000058; L29; YSS_RS09410				
sequence	MKYTEIKDKTAAELATMLKEKKVLLFTLKQKLKTMQLTNPKEISEVRKDIARINTAINALK* (61 aa)			
1*	RM4661 reference isoform	7034.48 Da	±0.00 Da	96.869 % (1516/1565)
2	MKYTEIKDKTAAELATMLKEKKVLLFTLKQKLKTMQLTNPKEISQVKKDIARINTAINALK* (E45Q + R47K)	7005.48 Da	-29.00 Da	2.428 % (38/1565)

RpmC/BACT000058; L29; YSS_RS09410				
3	MKYIEIKDKTAAELATMLKEKKVLLFTLKQKLKTMQLTNPKEISEVRKDIARINTAINALK* (T4I)	7046.53 Da	+12.05 Da	0.128 % (2/1565)
4	MKYTEIKDKTAAELATMLKEKKVLLFTLKQKLKTMQLTNPKEISQVKKDIARINTAINALR* (E45Q + R47K + K61R)	7033.49 Da	-0.99 Da	0.319 % (5/1565)
5	MKHTEIKDKTAAELATMLKEKKVLLFTLKQKLKTMQLTNPKEISEVRKDIARINTAINALK* (Y3H)	7008.44 Da	-26.04 Da	0.192 % (3/1565)
6*	MKYTEIKDKTAAELATMLKEKKVLLFTLKQKLKTMQLTNPKEISQAKKDIARINTAINALK* (E45Q+V46A+R47K)	6977.43 Da	-57,05 Da	new

RpmB/BACT000057; L28-M; YSS_RS02020				
sequence	(M)ARICQITGKGPMVGNNVSHANNKTKRRFLPNLRTVRVTLEDGTTRKMRIAASTLRTLKKQNSK* (63 aa)			
1*	RM4661 reference isoform	7078.30 Da	±0.00 Da	98.082 % (1534/1564)
2*	(M)ARICQITGKGPMVGNNVSHANNKTKRRFLPNLRTVRVTLEDGTTRKMRIAASTLRTLKKQNSN* (K64N)	7064.23 Da	-14.07 Da	1.662 % (26/1564)
3	(M)ARVCQITGKGPMVGNNVSHANNKTKRRFLPNLRTVRVTLEDGTTRKMRIAASTLRTLKKQNSK* (I4V)	7064.27 Da	-14.03 Da	0.128 % (2/1564)
4	(M)ARICQITGKGPMVGNNVSHANNKTKRRFLPNLRIVRVTLEDGTTRKMRIAASTLRTLKKQNSK* (T35I)	7090.36 Da	+12.06 Da	0,128 % (2/1564)
5*	(M)ARVCQVTGKGPMVGNNVSHANNKTKRRFLPNLRTVRVTLEDGTTRKMRIAASTLRTLKKQNSK* (I4V+I7V)	7050.25 Da	-28.05 Da	new

RpmI/BACT000064; L35-M; YSS_RS08275				
sequence	(M)PKMKSVKSAVKRFKVGKNKIKRGSAFRSHILTKKPAKRMRDLRTAKYVHSTNVKAVEKMLGI* (62 aa)			
1*	RM4661 reference isoform	7080.06 Da	±0.00 Da	99.616 % (1557/1563)
2	(M)PKMKSVKSAVKRFKVGKNKIKRGSAFRSHILTKKPAKRMRGLRTAKYVHSTNVKAVEKMLGI* (D42G)	7022.58 Da	-57.48 Da	0.320 % (5/1563)
3	(M)PKMKSVKSAVKRFKVGKNKIKRGSAFRSHILTKKPAKRMRNLRTAKYVHSTNVKAVEKMLGI* (D42N)	7079.64 Da	-0.42 Da	0.064 % (1/1563)

RpmE/BACT000060; L31; YSS_RS08510				
sequence	MKKEIHPEYVECKVSCACGNTFVTKSNKSELRVDICSSCHPFFTGSEKIVDAAGRVEKFKKKYAMQ* (66 aa)			
1*	RM4661 reference isoform	7463.68 Da	±0.00 Da	99.361 % (1555/1565)
2	MKKEIHPEYVECKVSCACGNTFVTKSNKSELRVDICSSCYPFFTGSEKIVDAAGRVEKFKK KYAMQ* (H40Y)	7489.72 Da	+26.04 Da	0.064 % (1/1565)
3	MKKEIHPEYVECKVSCACGNTFVTKSNKSELRVDICSSCHPFFTGSEKIVDAAGRVEKFKK KYSMQ* (A64S)	7479.68 Da	+16.00 Da	0.064 % (1/1565)
4	MKKEIHPEYVECKVSCACGNTFVTKSNKSELRVDICSSCHPFFTGSEKIVDAAGRVEKFKR KYAMQ* (K61R)	7491.69 Da	+28.01 Da	0.128 % (2/1565)
5	MKKEIHPEYVECKVSCACGNTFITKSNKSELRVDICSSCHPFFTGSEKIVDAAGRVEKFKK KYAMQ* (V23I)	7477.71 Da	+14.03 Da	0.192 % (3/1565)
6	MKKEIHPEYVECKVSCACGNTFVTKSNKSELRVDICSSCHPFFTGSEKIVDAAGRVEKFKK KYAM* (Q66STOP)	7335.55 Da	-128.13 Da	0.128 & (2/1565)
7	MKKEIHPEYVECKVSCACGNTFVTKSNKSELRVDICSSCHPFFTGSEKIIDAAGRVEKFKK KYAMQ* (V50I)	7477.71 Da	+14.03 Da	0.064 % (1/1565)

RplX/BACT000053; L24-M; YSS_RS09395				
sequence	(M)AVKLKIKKGDSVKVITGDDKGKTGKVLAVYPKTLKVVVEGCKIAKKAIKPSEKNPNGGFINKEMPMDISNVAKVQE* (77 aa)			
1*	RM4661 reference isoform	8151.75 Da	±0.00 Da	94.633 % (1481/1565)
2*	(M)AVKLKIKKGDSVKVITGDDKGKTGKVLAVYPKTLKVVVEGCKIAKKAIKPSEKNPNGG FMNKEMPMDISNVAKVQE* (I61M)	8169.78 Da	+18.03 Da	1.534 % (24/1565)
3*	(M)AVKLKIKKGDSVKVITGDEKGKTGKVLAVYPKTLKVVVEGCKIAKKAIKPSEKNPNGG FINKEMPMDISNVAKVQE* (D20E)	8165.78 Da	+14.03 Da	3.131 % (49/1565)
4	(M)AVKLKIKKGDSVKVITGDDKGKTGKVLAVYPKTLKVVVEGCKIAKKAIKPSEKNPNGG FINKEMPIDISNVAKVQE* (M67I)	8133.72 Da	-18.03 Da	0.192 % (3/1565)
5	(M)AVKLKIKKGDSVKIITGDDKGKTGKVLAVYPKTLKVVVEGCKIAKKAIKPSEKNPNGG FINKEMPMDISNVAKVQE* (V15I)	8165.78 Da	+14.03 Da	0.383 % (6/1565)
6	(M)AVKLKIKKGDSVKVITGDDKGKTGKVLAVYPKTLKVVVEGCKIAKKAIKPSEKNPNGG FINKV-PMDISNVAKVQE * (E64V)	8119.69 Da	-32,06 Da	0.064 % (1/1565)
7	(M)AVKLKIKKGDSVKVIIGDDKGKTGKVLAVYPKTLKVVVEGCKIAKKAIKPSEKNPNGG FINKEMPMDISNVAKVQE* (T17I)	8163.80 Da	+12.05 Da	0.064 % (1/1565)
8*	(M)AVKLKIKKGDSVKVITGDDKGKIGKVLAVYPKTLKVVVEGCKIAKKAIKPSEKNPNGG FINKEMPMDISNVAKVQE* (T24I)	8163.80 Da	+12.05 Da	new

RplX/BACT000053; L24-M; YSS_RS09395				
9*	(M)AVKLKIKKGDSVKIITGDDKGKTGKVLAVYPKTLKVVVEGCKIAKKAVKPSDKNPNGG FINKEMPMDISNVAKVQE* (V15I+I49V+E53D)	8137.72 Da	-32.06 Da	new

hypothetical protein DUF465 (Cj0449c homologue)				
sequence	MLHEYRELMSELKGKDAHFDKLFERHNELDDQIKDAEEGRNLLSDIEISNLKKEKLHIKDQLNQYLANYKK* (71 aa)			
1*	RM4661 reference isoform	8571.69 Da	±0.00 Da	89.317 % (1020/1142)
2*	MLHEYRELMSELKGKDAHFDKLFQRHNELDDQIKDAEEGRTPLSDLEISSLKKEKLHVKDE LNQYLANYKK* (E24Q + N41T + L42P + I46L + N50S + I58V + Q61E)	8501.59 Da	-70.10 Da	6.305 % (72/1142)
3	MLHEYRELMSELKGKDAHFDKLFERHNELDDQIKDAEEGRNLLSDIEISNLKKEKLYIKDQ LNQYLANYKK* (H47Y)	8597.72 Da	+26.03 Da	0.701 % (8/1142)
4*	MLHEYRELMSELKGKDAHFDKLFERHNELDDQIKDAEEGRALLSDIEISNLKKEKLHIKDE LNQYLSNYKK* (N41A + Q61E + A67S)	8545.65 Da	-26.04Da	3.240 % (37/1142)
5	MLHEYRELMSELKGKDAHFDKLFDRHNELDDMIKDAEEGRTSLSSMEISTLKKEKLHVKDE LSQYLANYKK* (E24D + Q32M + N41T + L42S + D45S + I46M + N50T + I58V + Q61E + N63S)	8458.60 Da	-113.09 Da	0.088 % (1/1142)
6	MLHEYRELMSELKGKDAHFDKLFERHNELDDQIKDAEEGRNLLSDIEISNLKKEKLHIKDR LNQYLANYKK* (Q61R)	8599.74 Da	+28.05 Da	0.088 % (1/1142)
7*	MLHEYRELISELKGKDAHFDKLFERHNELDDQIKDAEEGRNLLSDIEISNLKKEKLHIKDQ LNQYLANYKK* (M9I)	8553.65 Da	-18.04 Da	0.175 % (2/1142)
8	MLHEYRELMSELKGKDAYFDKLFERHNELDDQIKDAEEGRNLLSDIEISNLKKEKLHIKDQ LNQYLANYKK* (H18Y)	8597.72 Da	+26.03 Da	0.088 % (1/1142)

RpsP/BACT000016; S16; YSS_RS03245				
sequence	MTVIRLTRMGRTKRPFYRIVVTDSRKRRDGGWIESIGYYNPMVEPEVVKFDSERLAYWKSVGAKLSDKVASITSK* (75 aa)			
1 *	RM4661 reference isoform	8728.16 Da	±0.00 Da	0.985 % (1542/1565)
2	MTVIRLTRMGRTKRPFYRIVVTDSRKRRDGGWIESIGYYNPMVEPEVVKFDSERLAYWKSV GAKLSDKVASIISK * (T73I)	8740.21 Da	+12.05 Da	0.383 % (6/1565)
3	MTVIRLTRMGRTKRPFYRIVVTDSRKRRDGGWIESIGYYNPMVEPEVVKFDPERLAYWKSV GAKLSDKVASITSK * (S52P)	8738.20 Da	+10.04 Da	0.064 % (1/1565)
4	MTVIRLTRMGRTKRPFYRIVVTDSRKRRDGGWIESIGYYNPMVEPEVVKFDSERLAYWKSV GAKLSDKVASITNK * (S74N)	8755.18 Da	+27.02 Da	0.191 % (5/1565)
5	MTIIRLTRMGRTKRPFYRIVVTDSRKRRDGGWIESIGYYNPMVEPEVVKFDSERLAYWKSV	8742.18 Da	+14.02 Da	0.192 % (3/1565)

RpsP/BACT000016; S16; YSS_RS03245				
	GAKLSDKVASITSK* (V3I)			
6	MTVIRLTRMGRIKRPFYRIVVTDSRKRRDGGWIESIGYYNPMVEPEVVKFDSERLAYWKSV<br>GAKLSDKVASITSK* (T12I)	8740.21 Da	12.05 Da	0.192 % (3/1565)
7	MTVIRLTRMGRTKRPFYRIVVTDSRKRRDGGWIESIGYYNPMVKPEVVKFDSERLAYWKSV<br>GAKLSDKVASITSK * (E44K)	8727.22 Da	-0.94 Da	0.128 % (2/1565)
8	MTVIRLTRMGRTKRPFYRIVVTDSRKRRDGGWIESIGYYNPMVDPEVVKFDSERLAYWKSV<br>GAKLSDKVASITSK * (E44D)	8714.13 Da	-14.03 Da	0.064 % (1/1565)

RpmA/BACT000056; L27-M; YSS_RS00440				
sequence	(M)AHKKGQGSTQNNRDSIGRRLGVKKFGGEFVRAGNIIIRQRGTATHAGNNVGIGKDHTIFALIDGFVKFERKDKDRKKVSVYPA* (84 aa)			
1*	RM4661 reference isoform	9135.42 Da	±0.00 Da	93.482 % (1463/1565)
2	(M)AHKKGQGSTQNNRDSIGRRLGVKKFGGEFVRAGNIIIRQRGTATHAGNNVGIGKDHTI<br>FALIDGFVKFERKDKDRKKVSVYPV* (A84V)	9163.47 Da	+28.05 Da	3.323 % (52/1565)
3*	(M)AHKKGQGSTQNNRDSIGRRLGVKKFGGEFVRAGNIIIRQRGTATHAGNNVGMGKDHTI<br>FALIDGFVKFERKDKDRKKVSVYPA * (M53I)	9153.45 Da	+18.03 Da	3.131 % (49/1565)
4	(M)AHKKGQGSTQNNRDSIGRRLGVKKFGGEFVRAGNIIIRQRGTATHAGNNVGIGKDHTI<br>FALIDGFVKFERKDKDREKVSVYPA * (K77E)	9136.36 Da	+0.94 Da	0.064 % (1/1565)

RpsT/BACT000020; S20-M; YSS_RS00790				
sequence	(M)ANHKSAEKRARQTIKKTERNRFYRTRLKNITKAVREAAAKNDKNAANEALKIANKSIHAMVSRGFLKKQTASRRVSRLALLVNKIA* (86 aa)			
1	RM4661 reference isoform	9771.42 Da	±0.00 Da	94.633 % (1481/1565)
2*	(M)ANHKSAEKRARQTIKKTERNRFYRTRLKNITKAVREAAAKNDKNAANEALKIANKSIH<br>AMASRGFLKKQTASRRVSRLALLVNKIA* (V62A)	9743.37 Da	-37.05 Da	3.323 % (52/1565)
3	(M)ANHKSAEKRARQTIKKTERNRFYRTRLKNITKAVREAAAQNDKNAANEALKIANKGIH<br>AMVSRGFLKKQTASRRVSRLALLVNKIA* (K41Q+S57G)	9741.35 Da	-30.07 Da	0.192 % (3/1565)
4*	(M)ANHKSAEKRARQTIKKTERNRFYRTRLKNITKAVREAAAQNDKNAANEALKIANKSIH<br>AMVSRGFLKKQTASRRVSRLALLVNKIA* (K41Q)	9771.38 Da	-0.03 Da	1.661 % (26/1565)
5	(M)ANHKSAEKRARQTIKKTERNRFYRTRLKNITKAVREAAAKNNKNAANEALKIANKSIH<br>AMVSRGFLKKQTASRRVSRLALLVNKIA* (D43N)	9770.44 Da	-0.98 Da	0.128 % (2/1565)

RpsT/BACT000020; S20-M; YSS_RS00790				
6	(M)ANHKSAEKRARQTIKKTERNRFYRTRLKNITKAVREAAAKNDKNATNEALKIANKSIH AMVSRGFLKKQTASRRVSRLALLVNKIA* (A47T)	9801.45 Da	+30.03 Da	0.128 % (2/1565)
7	(M)ANHKSAEKRARQTIKKTERNRFYRTRLKNITKAVREAAAKNDKNSANEALKIANKSIH AMVSRGFLKKQTASRRVSRLALLVNKIA* (A46S)	9787.42 Da	+16.00 Da	0.128 % (2/1565)
8*	(M)ANHKSSEKRARQTIKKTERNRFYRTRLKNITKAVREAAQNNDKAAATEALKVANKSIH AMVSRGFLKKQTASRRVSRLALLVNKIA* (A7S+A40Q+N48T+I53V)	9760.35 Da	-11.07 Da	new

RpsO/BACT000015; S15-M; YSS_RS04540				
sequence	(M)ALDSAKKAEIVAKFARKAGDTGSTEVQVALLSARITELTEHLKIYKKDFSSRLGLLKLVGQRKRLLAYLKRKDYNSYSKLITELNLRDK* (89 aa)			
1*	RM4661 reference isoform	10096.83 Da	±0.00 Da	% (16/1555)
2*	(M)ALDSAKKAEIVAKFARKAGDTGSTEVQVALLTARIAELTEHLKIYKKDFSSRLGLLKL VGQRKRLLAYLKRKDYNSYSKLITELNLRDK* (S33T + T37A)	10080.83 Da	-16.00 Da	% (75/1555)
3*	(M)ALDSAKKAEIVAKFARKAGDTGSTEVQVALLSARIAELTEHLKIYKKDFSSRLGLLKL VGQRKRLLAYLKRKDYNSYSKLITELNLRDK* (T37A)	10066.80 Da	-30.03 Da	% (1453/1555)
4	(M)ALDSAKKAEIVAKFARKAGDTGSTEVQVALLSTRIAELTEHLKIYKKDFSSRLGLLKL VGQRKRLLAYLKRKDYNSYSKLITELNLRDK* (A34T + T37A)	10096.83 Da	±0.00 Da	% (1/1555)
5	(M)ALDSAKKAEIVAKFAKKPGDTGSTEVQVALLTARIAELTEHLKIYKKDFSSRLGLLKL VGQRKRLLAYLKRKDYNSYSKLITELNLRDK* (R17K + A19P + S33T + T37A)	10078.85 Da	-17.98 Da	% (4/1555)
6	(M)ALDSAKKAEIVAKFARKASDTGSTEVQVALLSARIAELTEHLKIYKKDFSSRLGLLKL VGQRKRLLAYLKRKDYNSYSKLITELNLRDK* (G20S + T37A)	10096.83 Da	±0.00 Da	% (2/1555)
7	(M)ALDSAKKAEIVAKFAKKPGDTGSTEVQVALLTARIAELTEHLKIYKKDFSSRLGLLKL VGQRKRLLSYLKRKDYNSYSKLITELNLRDK* (R17K + A19P + S33T + T37A+ A68S)	10094.85 Da	-1.98 Da	% (2/1555)
8	(M)ALDSAKKAEIVAKFARKAGDTGSTEVQVALLSARIAELTEHLKIYKKDFSSRLGLLKL VGQRKRLLAYLKRKDYNSYSKLIAELNLRDK* (T37A + T83A)	10030.68 Da	-66.15 Da	% (1/1555)
9	(M)ALDSAKKAEIVAKFARKAGDTGSTEVQVALLSARIAELTEHLKIYKKDFSSRLGLLKL VGQRKRLLAYLKRKDYNSYGKLITELNLRDK* (T37A + S79G)	10030.68 Da	-66,15 Da	% (1/1555)

RpsS/BACT000019; S19-M; YSS_RS09430				
sequence	(M)ARSLKKGPFVDDHVMKKVIAAKKANDNKPIKTWSRRSTIIPDMIGLTFNVHNGKSFIPVYITENHIGYKLGEFAPTRTFKGHKGSVQKKIGK* (92 aa)			
1 *	RM4661 reference isoform	10334.13 Da	±0.00 Da	97.636 % (1528/1565)

RpsS/BACT000019; S19-M; YSS_RS09430				
2	(M)ARSLKKGPFVDDHVMKKVIAAKKANDNKPIKTWSRRSTITPDMIGLTFNVHNGKSFIP VYITENHIGYKLGEFAPTRTFKGHKGSVQKKIGK* (I41T)	10322.08 Da	-12.05 Da	1.917 % (30/1565)
3	(M)ARSLKKGPFVDDHVMKKVIAAKEANDNKPIKTWSRRSTIIPDMIGLTFNVHNGKSFIP VYITENHIGYKLGEFAPTRTFKGHKGSVQKKIGK* (K24E)	10335.07 Da	+0.94 Da	0.319 % (5/1565)
4	(M)ARSLKKGPFVDDHVIKKVIAAKKANDNKPIKTWSRRSTIIPDMIGLTFNVHNGKSFIP VYITENHIGYKLGEFAPTRTFKGHKGSVQKKIGK* (M16I)	10316.10 Da	-18.03 Da	0.128 % (2/1565)

Legend:
* observed in test population
AA numbering including start-methionine, if mass spectrometry indicates its absence it is written in brackets (M)

Supplementary Table 3. Comparison of measured and calculated average and monoisotopic biomarker masses

Biomarker	Isoform	Measured Mass [Da]	Standard deviation	Δ Measured mass/ Average mass	Monoisotopic mass [Da]	Average Mass [Da]
L36	Isoform 1	4365	0.388	0.61	4361.43	4364.39
L36	Isoform 3	4378	0.191	0.41	4375.45	4378.41
L34	Isoform 1	5245	0.405	0.72	5505.99	5244.28
L32-M	Isoform 1	5510	0.365	0.55	5505.99	5509.45
L33	Isoform 1	6127	0.528	0.73	6122.28	6126.27
L33	Isoform 2	6155	0.781	1.29	6152.29	6156.29
L33	Isoform 5	6143	0.385	0.73	6138.27	6142.27
S14-M	Isoform 1	6810	0.586	1.24	6806.67	6811.24
S14-M	Isoform 4	6784	0.485	0.17	6779.63	6784.17
L29	Isoform 1	7035	0.484	0.52	7029.99	7034.48
L29	Isoform 6	6977	0.358	0.43	6972.97	6977.43
L28-M	Isoform 1	7078	0.427	0.30	7073.91	7078.30
L28-M	Isoform 2	7065	0.042	0.77	7059.86	7064.23
L28-M	Isoform 5	7050	0.473	0.25	7045.88	7050.25
L35-M	Isoform 1	7080	0.422	0.06	7076.11	7080.06
L31	Isoform 1	7463	0.488	0.68	7458.66	7463.68
L24-M	Isoform 1	8151	0.295	0.75	8164.54	8151.75
L24-M	Isoform 2	8169	0.369	0.78	8146.58	8169.78
L24-M	Isoform 3	8165	0.166	0.78	8160.60	8165.78
L24-M	Isoform 8	8163	0.142	0.80	8158,62	8163.80
L24-M	Isoform 9	8137	0.361	0.72	8132.57	8137.72
HypProt	Isoform 1	8572	0.412	0.31	8566.38	8571.69
HypProt	Isoform 2	8502	0.224	0.41	8496.33	8501.59
HypProt	Isoform 4	8546	0.599	0.35	8540.35	8545.65
HypProt	Isoform 7	8555	0.391	1.35	8548.42	8553.65
S16	Isoform 1	8729	0.231	0.84	8722.64	8728.16
L27-M	Isoform 1	9136	0.438	0.58	9147.90	9135.42
L27-M	Isoform 3	9154	0.295	0.55	9129.95	9153.45
S20-M	Isoform 2	9743	0.459	0.37	9765.55	9743.37
S20-M	Isoform 4	9772	0.101	0.62	9765.51	9771.38
S20-M	Isoform 8	9760	0.188	0.35	9754.50	9760.35
S15-M	Isoform 1	1096	0.363	0.83	10090.71	10096.83
S15-M	Isoform 2	1080	0.397	0.83	10074.71	10080.83
S15-M	Isoform 3	1066	0.620	0.80	10060.70	10066.80
S19-M	Isoform 1	10335	0.715	0.87	10327.66	10334.13

The measured biomarker masses and the corresponding standard deviations are based on six measurements. The molecular weight calculator in the ExPASy

Bioinformatics Resource Portal (http://web.expasy.org/compute_pi/) was used to calculate average and monoisotopic biomarker masses.

Supplementary Table 4. Listing of the Accession Numbers of the identified allelic isoforms of the biomarkers

Biomarker	Isoform	Gene Accession	Protein Accession
L36	1	CP018900.1	APT19528.1
L36	3	MK270367	MK270367
L34	1	CP028187.1	AVS39538.1
L32-M	1	CP004066.1	AGV10487.1
L33	1	CP028187.1	AVS38169.1
L33	2	CP023545.1	ATG67527.1
L33	5	CP024136.1	WP_020975142.1
S14-M	1	CP007181.1	AHK77725.1
S14-M	4	MK270368	MK270368
L29	1	CP025281.1	AUG27826.1
L29	6	MK270369	MK270369
L28-M	1	CP028187.1	AVS38189.1
L28-M	2	CP028187.1	WP_020975162.1
L28-M	5	MK270370	MK270370
L35-M	1	CP007181.1	AHK77513.1
L31	1	CP028187.1	AVS38768.1
L24-M	1	CP007181.1	AHK77727.1
L24-M	2	CP025281.1	AUG27823.1
L24-M	3	CP017875.1	APA58854.1
L24-M	8	MK270372	MK270372
L24-M	9	MK270371	MK270371
HypProt	1	CP028187.1	AVS38190.1
HypProt	2	CP025281.1	WP_020975163.1
HypProt	4	CP025283.1	WP_038838694.1
HypProt	7	CP017873.1	WP_002831949.1
S16	1	CP028187.1	WP_002776499.1
L27-M	1	CP018900.1	APT19449.1
L27-M	3	CP023545.1	ATG66444.1
S20-M	1	CP017025.1	AOH49294.1
S20-M	2	CP028187.1	AVS38483.1
S20-M	4	HG326877.1	CDG56774.1
S20-M	8	MK270374	MK270374
S15-M	1	CP007181.1	AHK76813.1
S15-M	2	CP017878.1	APA61584.1
S15-M	3	CP028187.1	AVS39511.1
S19-M	1	CP017025.1	AOH50892.1

The accession numbers for all detected biomarkers isoforms both for the gene/nucleotide sequence and for the amino acid sequence are given in the table below.

Supplementary Figures

Supplementary Figure 1. MLST based neighbor-joining dendrogram showing the different clades of *C. coli* and *C. jejuni.*

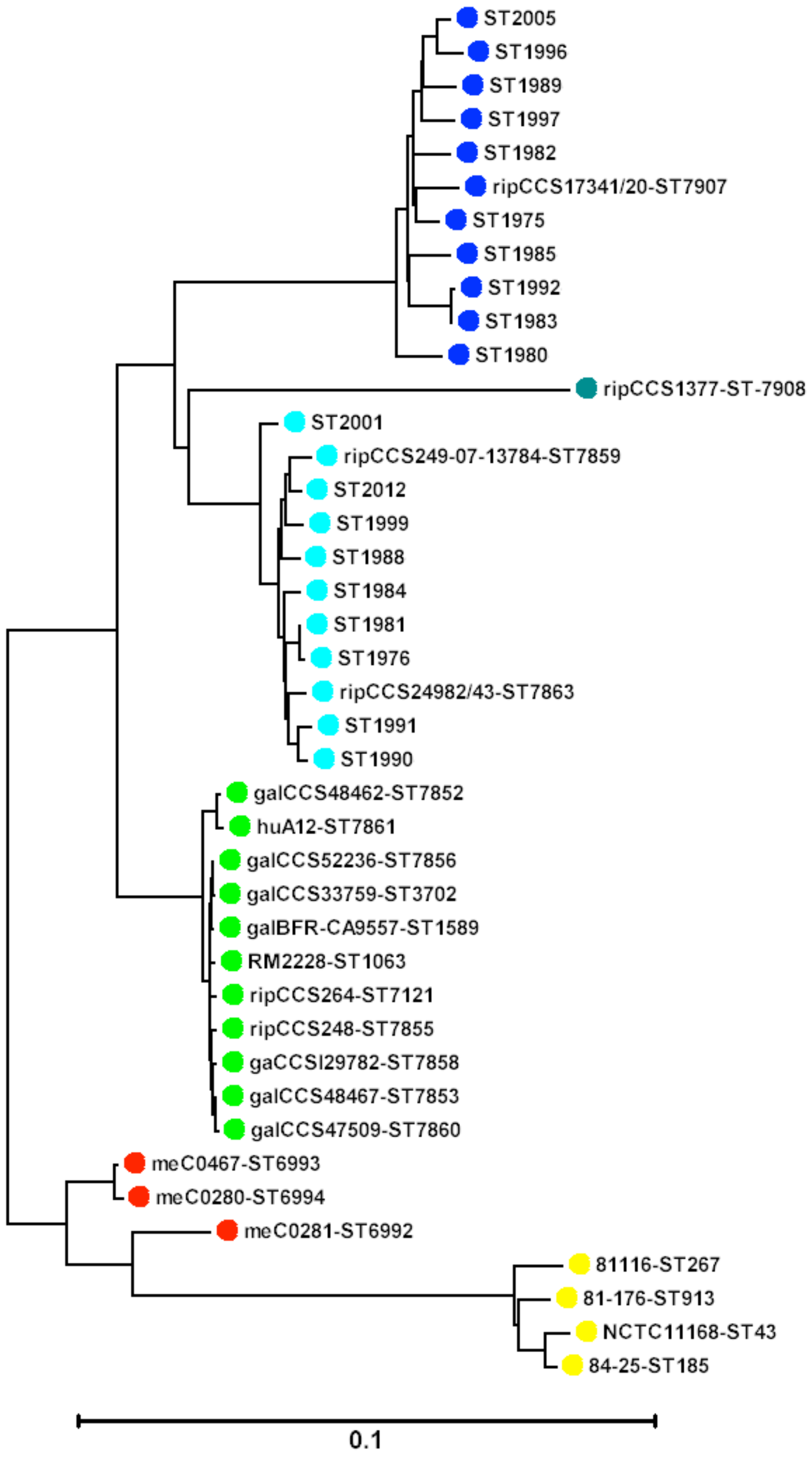

The depicted MLST-based neighbor-joining tree shows the phylogenetic relationship of the isolates CCS1377, meC0280, mecC0281, and meC0467 in relation to reference strains representing the established three *C. coli* clades and *C. jejuni*. The evolutionary history was inferred using the Neighbor-joining method[57]. The optimal tree with the sum of branch length = 0.509 is shown. The tree is drawn to scale, with branch lengths in the same units as those of the evolutionary distances used to infer the phylogenetic tree. The evolutionary distances were computed using the Maximum Composite Likelihood method[58] and are in the units of the number of base substitutions per site. Codon positions included were 1st+2nd+3rd+Noncoding. Evolutionary analyses were conducted in MEGA7[55]. Color codes: clade 1 – green, clade 2 – light blue, clade 3 – dark blue, isolate CCS1377 – dark green purple, isolates meC0280, mecC0281, and meC0467 – red, *C. jejuni* strains - yellow.

Supplementary Figure 2. Phylogenetic relationship between *C. coli* and *C. jejuni* ssp. *jejuni* isolates.

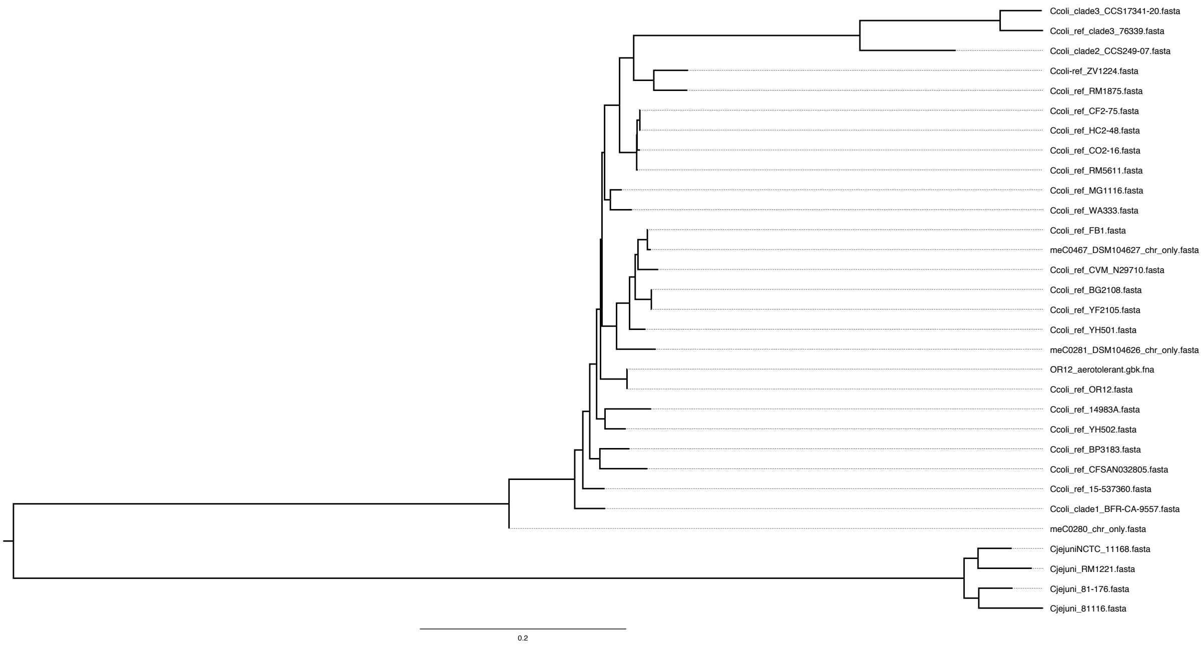

Core-genome alignments were derived from a set of 31 chromosomal genome sequences by using Parsnp. FastTree2[59] was used to calculate the maximum-likelihood (ML) phylogenetic tree. Parsnp and FastTree2 are both implemented in the Harvest package[60]. The ML tree includes 24 chromosomal genome sequences of *C. coli* isolates currently available in GenBank. In addition, the chromosomal genome sequences of meC0280, mecC0281, and meC0467 as well as four *C. jejuni* reference chromosomal genomes sequences have been included in the tree.
